# Supplementary material for: RopB represses the transcription of speB in the absence of SIP in group A Streptococcus
Source: Life Sci Alliance. 2023 Mar 31;6(6):e202201809. doi: 10.26508/lsa.202201809 (PMC10071013; doi:10.26508/lsa.202201809)

Fig. 1B

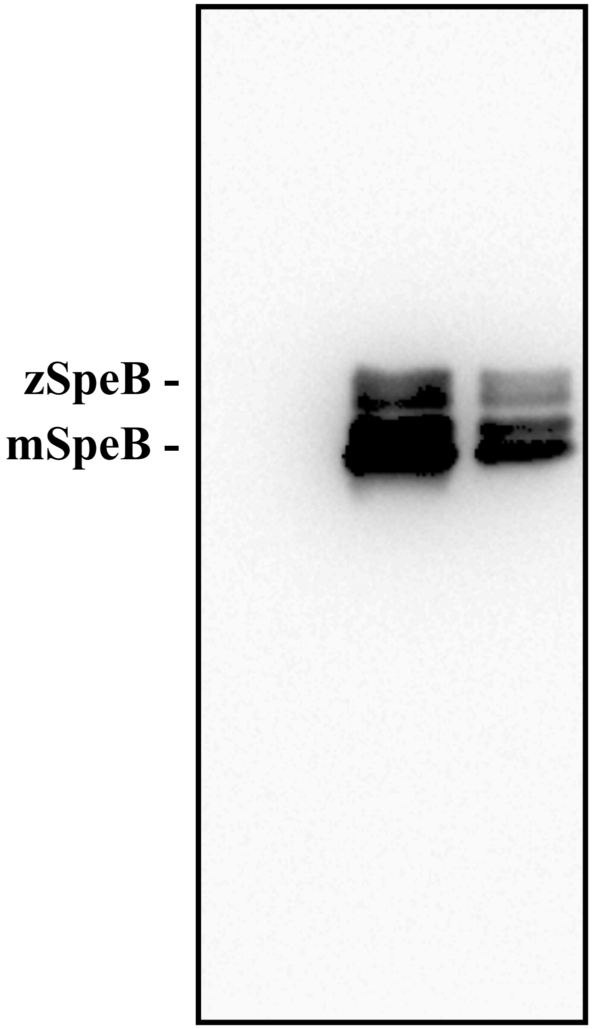

Fig. 1E (the upper panel)

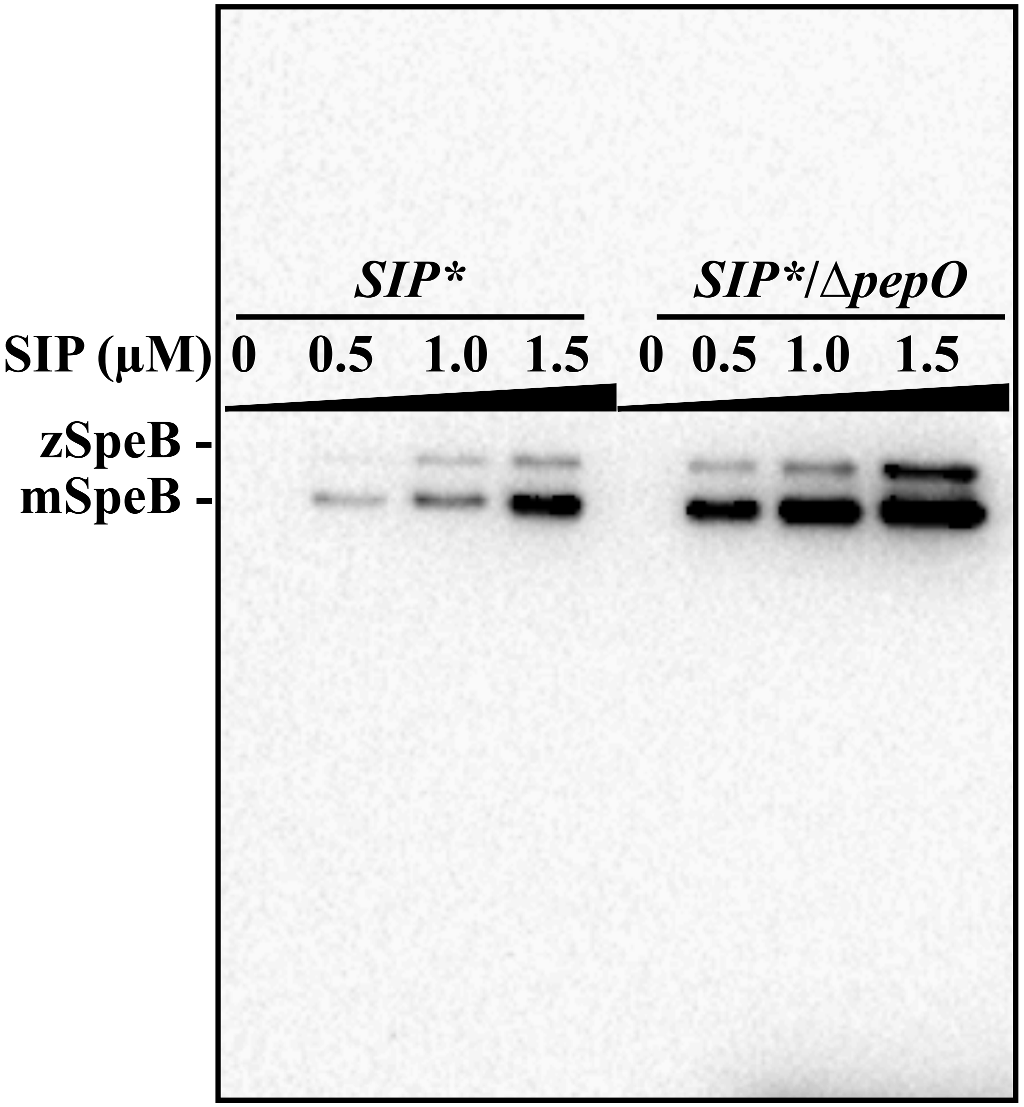

Fig. 1E (the lower panel)

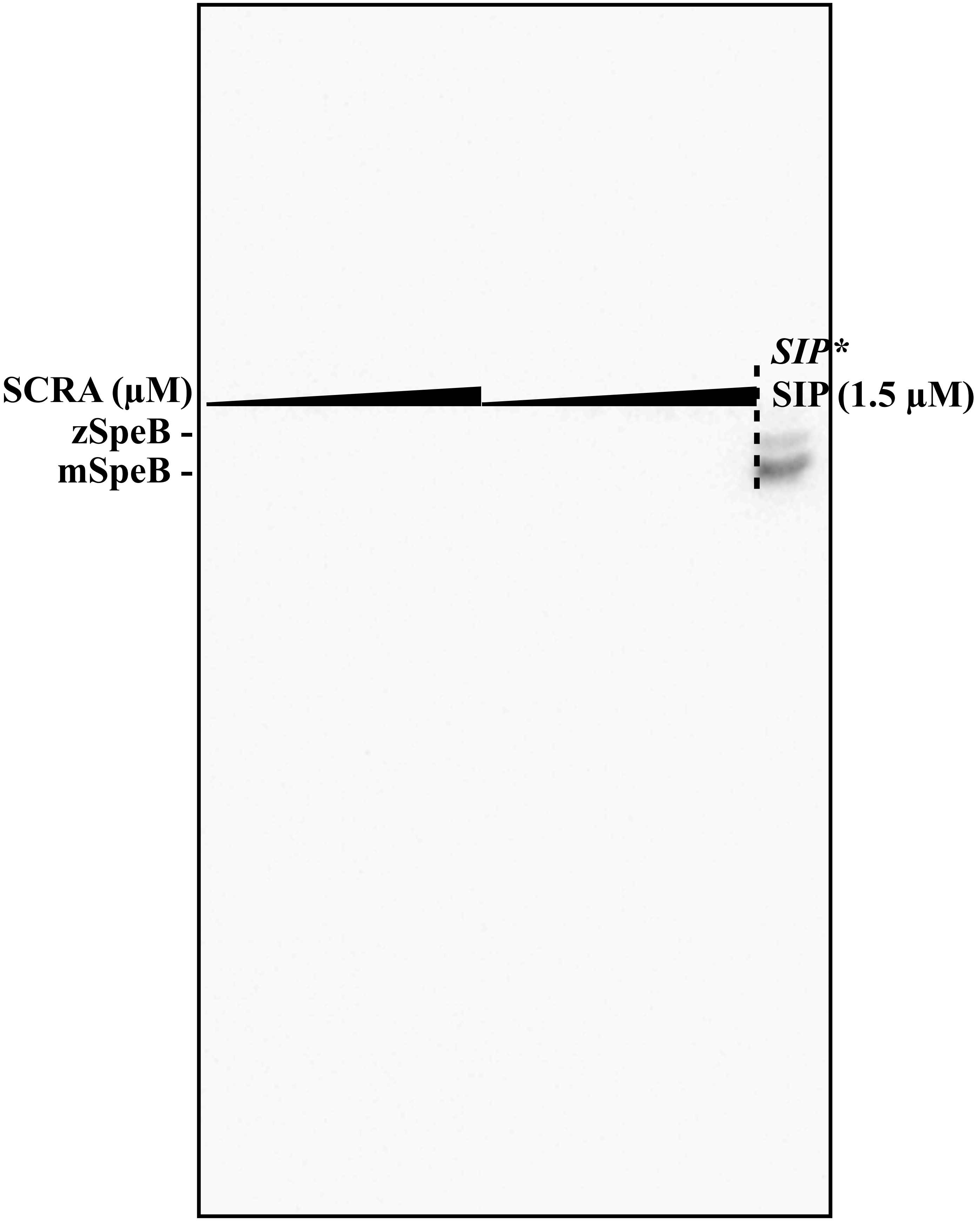

Fig. 1F

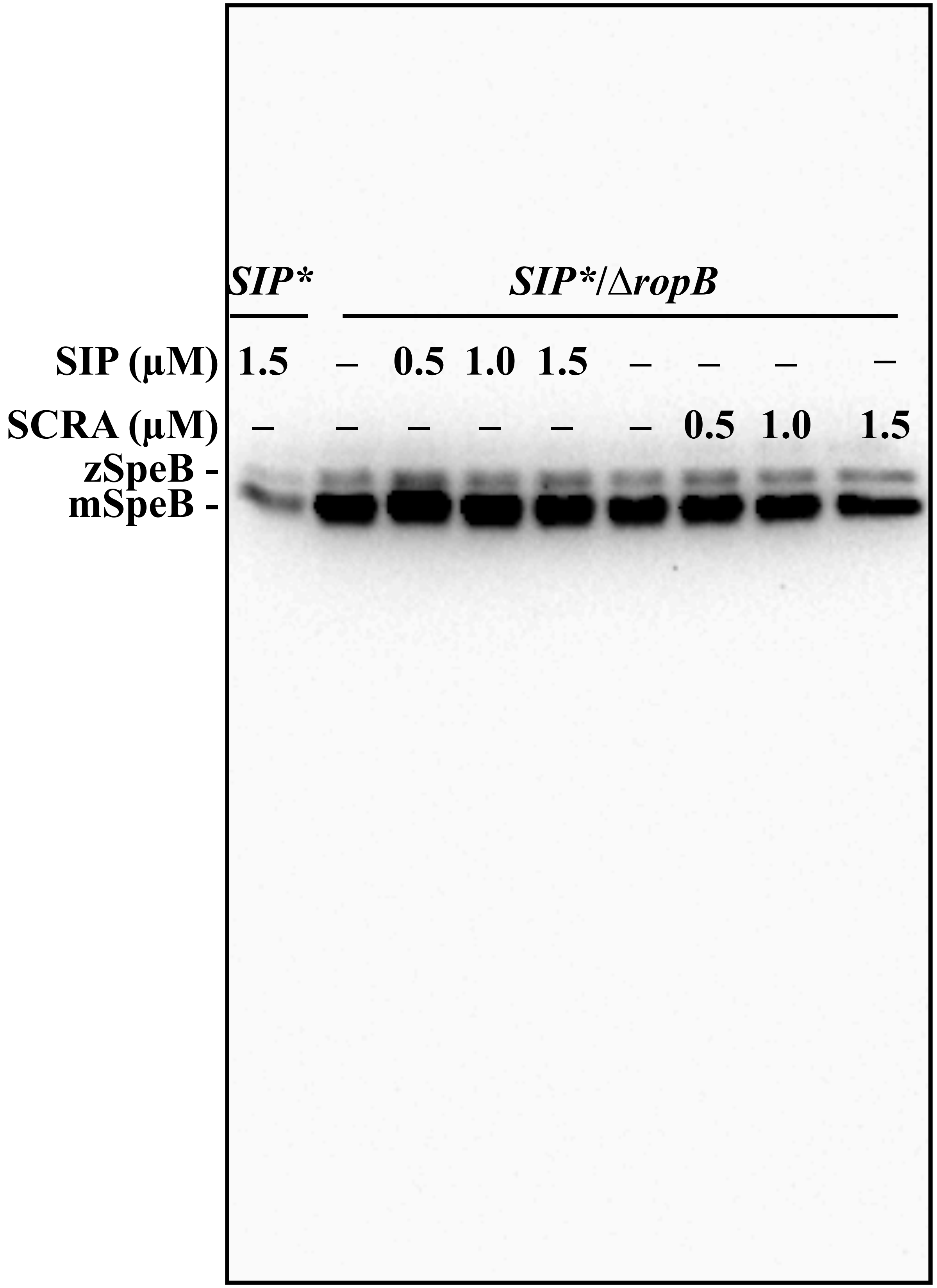

Fig. 1H

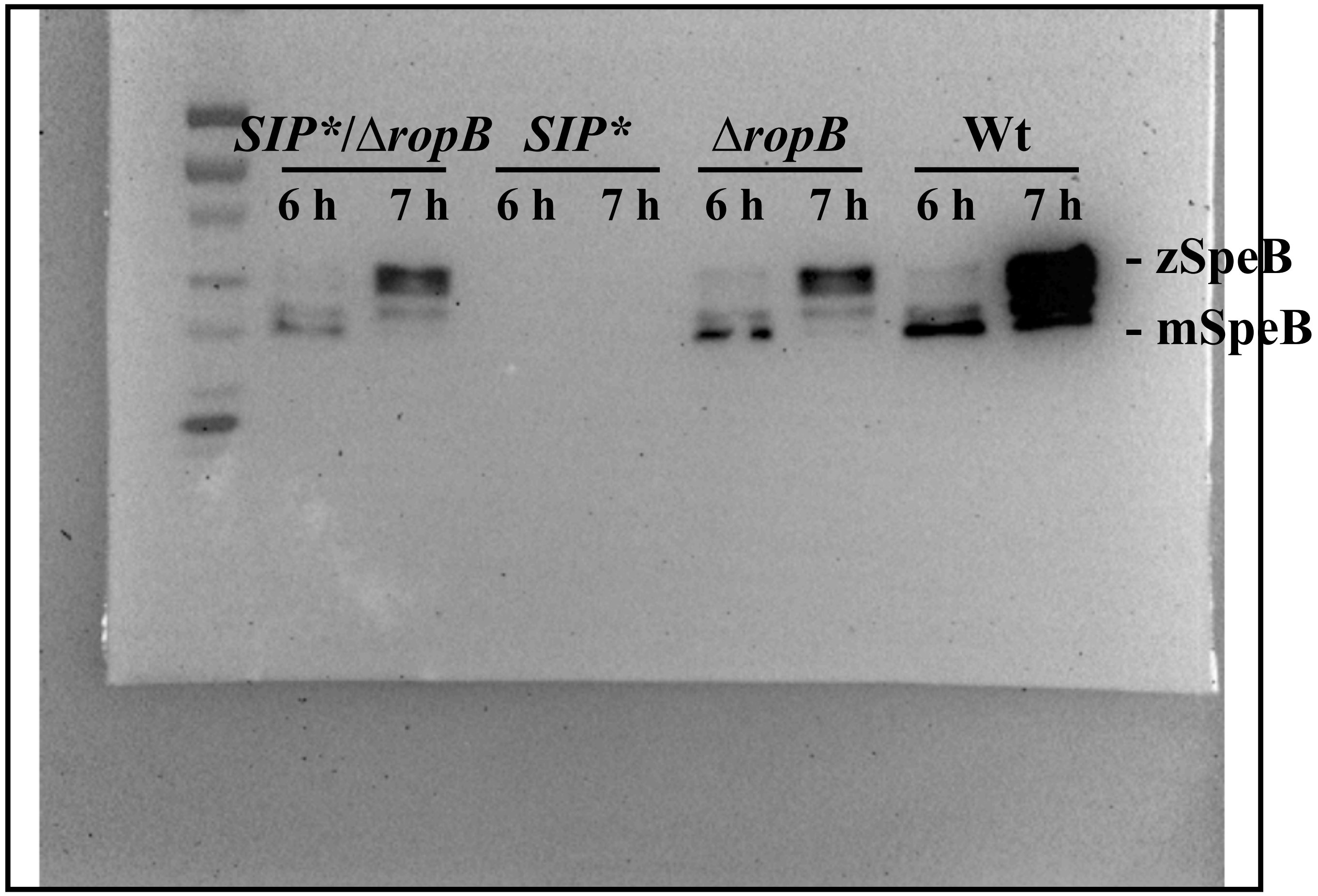

Fig. 2C

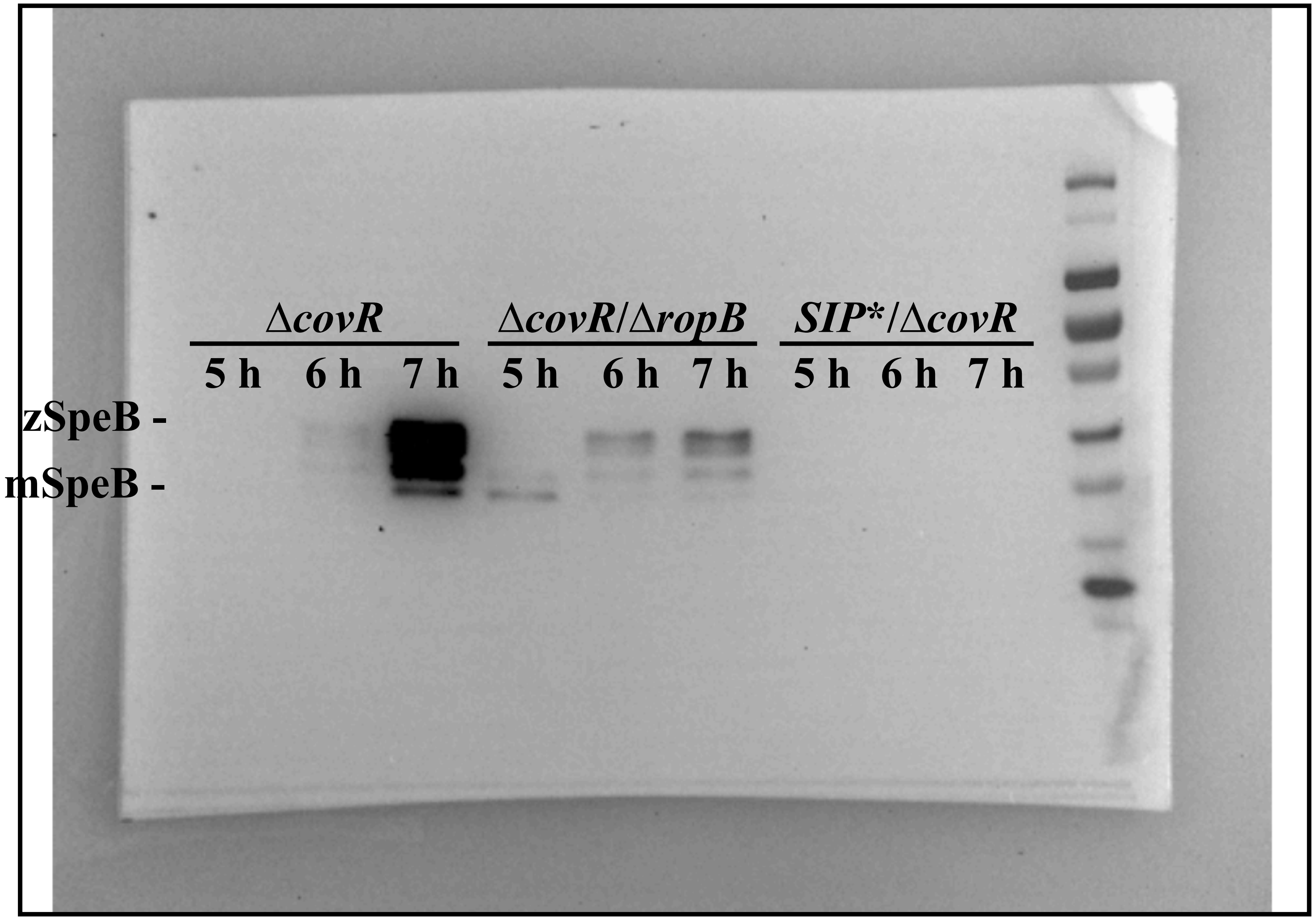

ΔcovR
ΔcovR/ΔropB
SIP*/ΔcovR
5 h
6 h
7 h
5 h
6 h
7 h
5 h
6 h
7 h
zSpeB -
mSpeB -

Fig. 2E

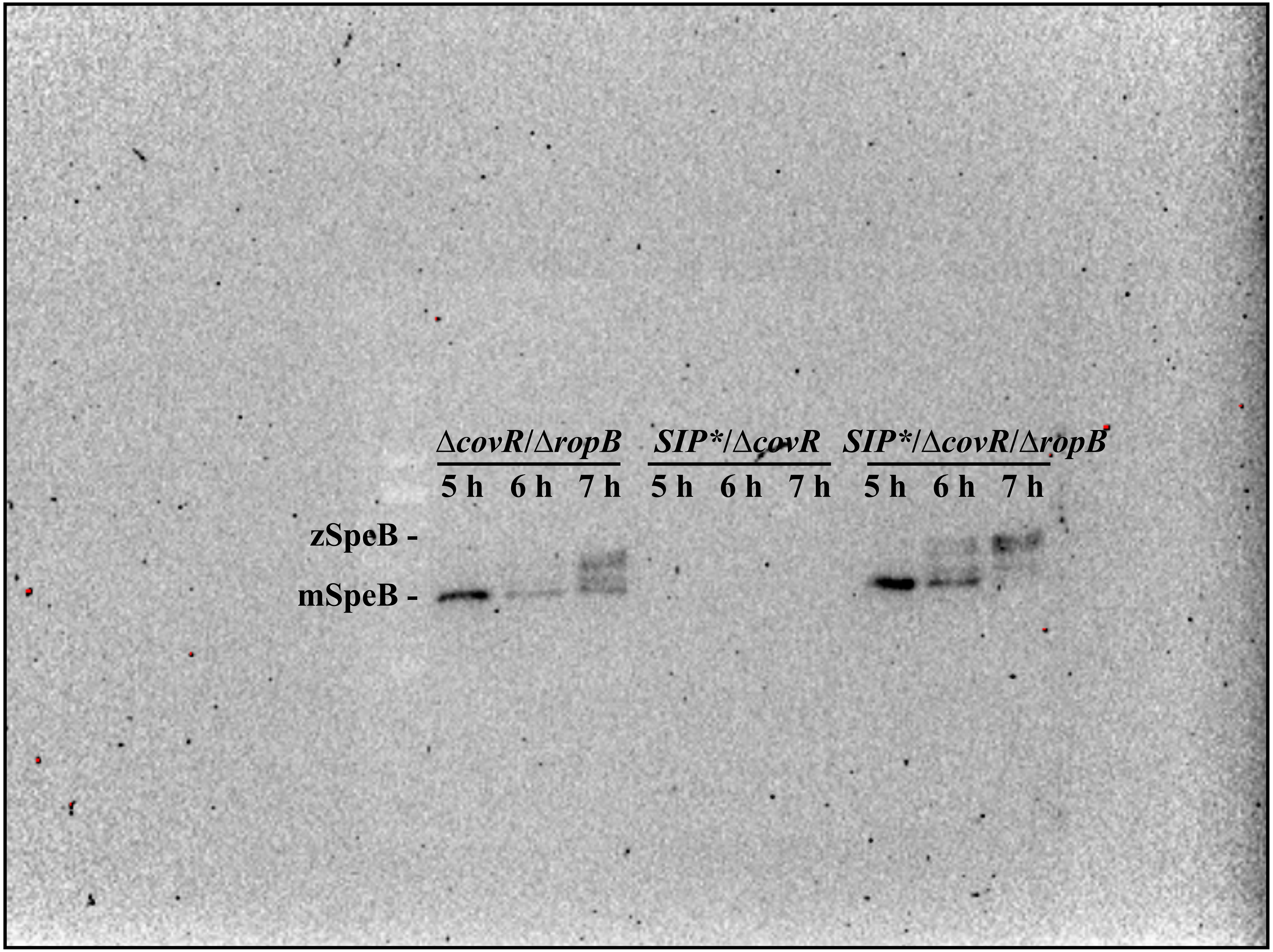
ΔcovR/ΔropB
SIP*/ΔcovR
SIP*/ΔcovR/ΔropB
5 h 6 h 7 h 5 h 6 h 7 h 5 h 6 h 7 h
zSpeB -
mSpeB -

Fig. 3A

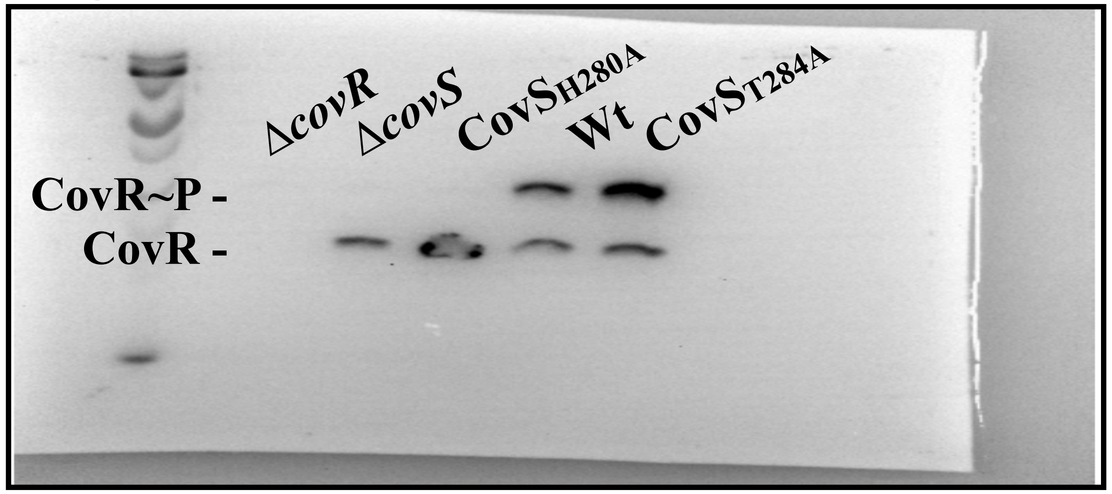

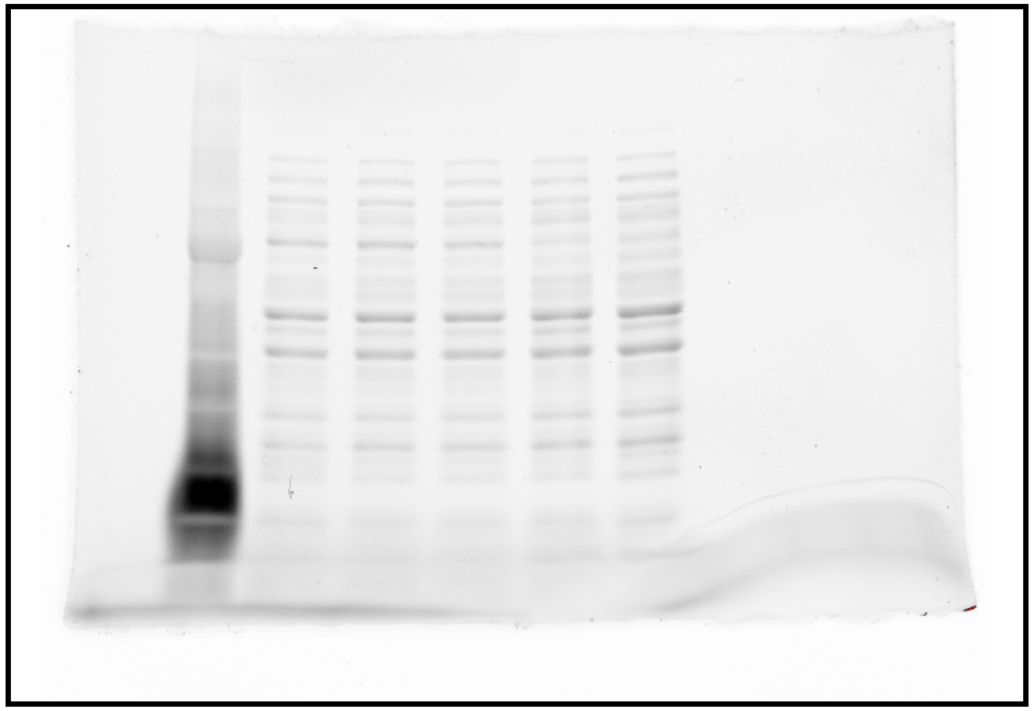

Fig. 3B

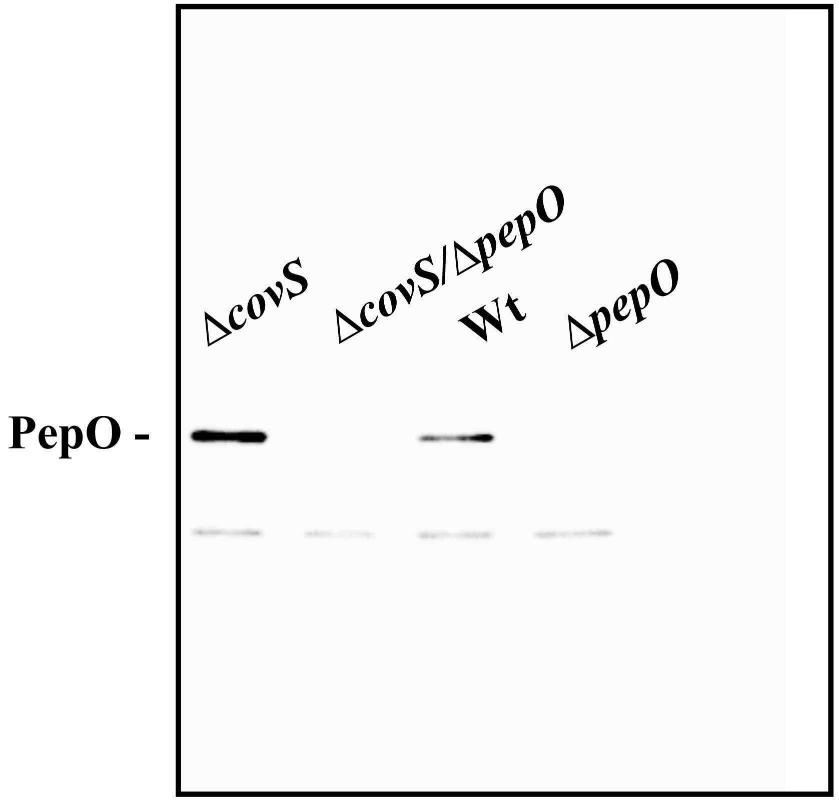

Total protein

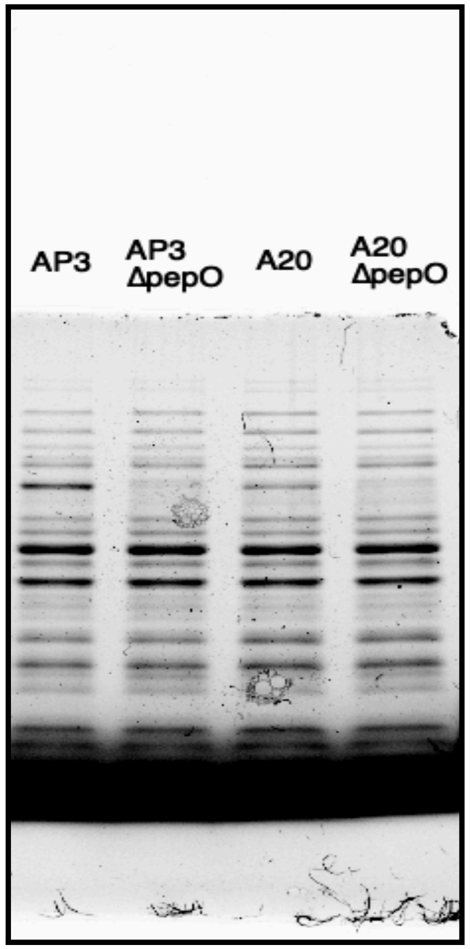

Fig. 3E

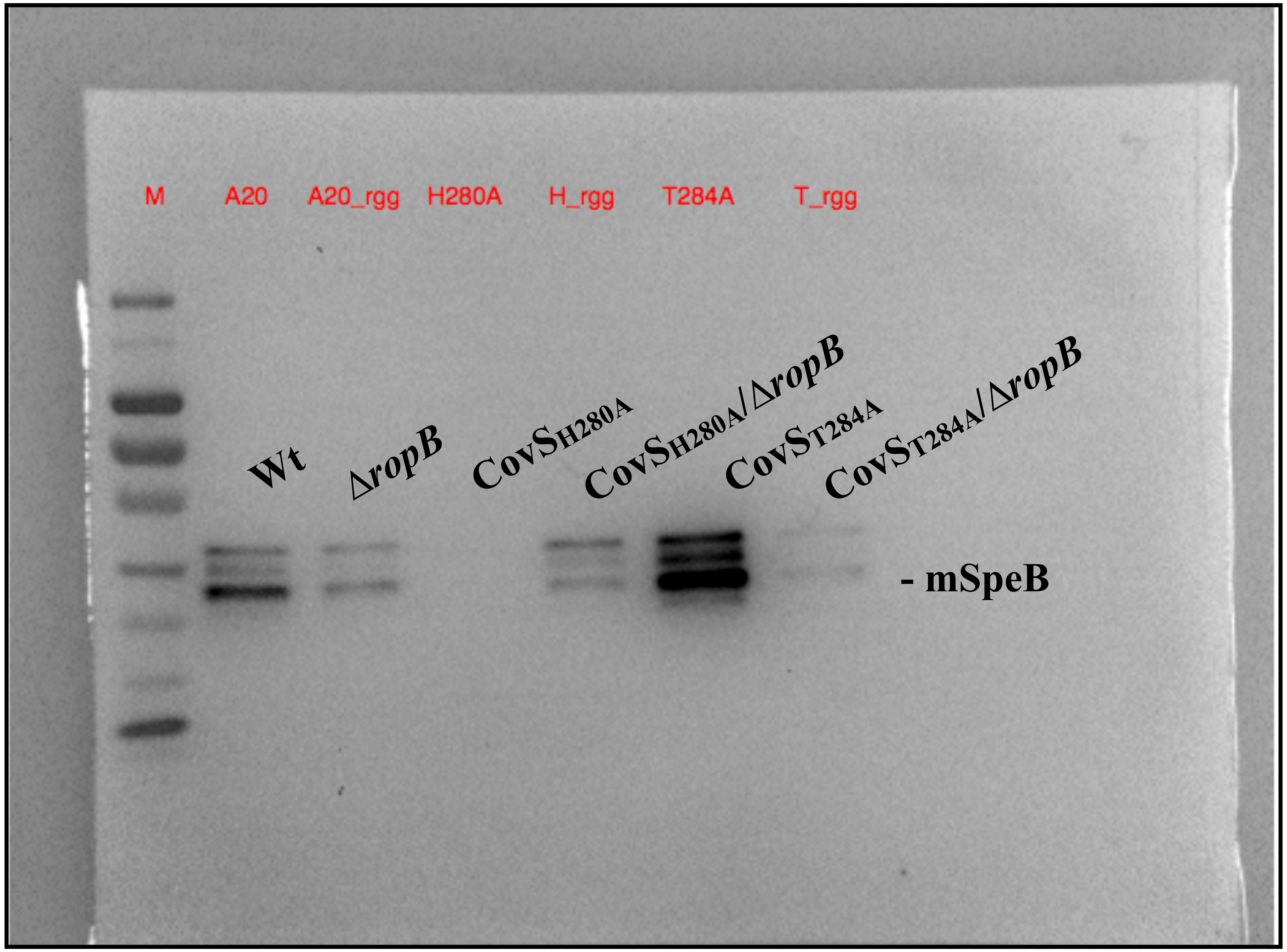
M
A20
A20_rgg
H280A
H_rgg
T284A
T_rgg
Wt
ΔropB
CovSH280A
CovSH280A/ΔropB
CovST284A
CovST284A/ΔropB
- mSpeB

Supplementary Fig. S4B

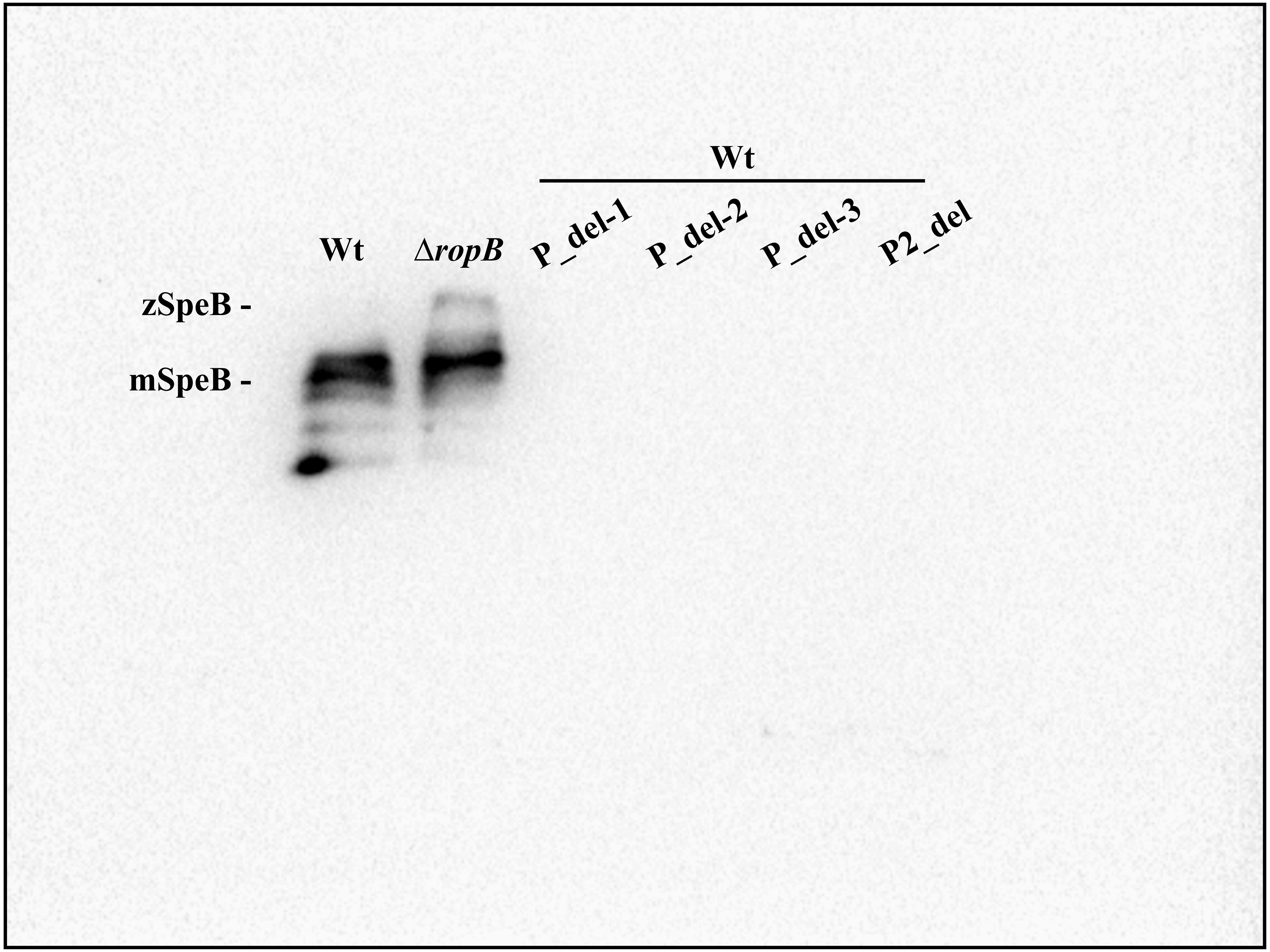

Supplement: Supplementary file 1 [file LSA-2022-01809_SdataF1.1_F2.1_F3.1_FS4.pdf]
