# Supplementary material for: RopB represses the transcription of speB in the absence of SIP in group A Streptococcus
Source: Life Sci Alliance. 2023 Mar 31;6(6):e202201809. doi: 10.26508/lsa.202201809 (PMC10071013; doi:10.26508/lsa.202201809)
Supplement: Supplementary file 2 [file LSA-2022-01809_SdataF1.2_F2.2_F3.2_F4_F5_FS2_FS3.pdf]

Fig 1A	A20	ΔpepO	SIP*
	1	4.599	0.001
	0.895	4.857	0.001
	1.000	4.991	0.000
	1.041	5.273	0.000
Fig 1A	A20	ΔpepO	SIP*
	1.02	4.67	0.00
	0.91	4.94	0.00
	1.02	5.07	0.00
	1.06	5.36	0.00
Mean	1.00	5.01	0.00
StdEv	0.06	0.29	0.00

Fig. 1B	SIP* (OD600=1)		
SpeB	Vec	Prgg-SIP	PspeB (SIP)
	1.00	4040.00	1675.00
	1.05	3848.00	1652.00
	1.00	6794.00	1710.00
	1.12	6841.00	2006.00
SpeB	Vec	Prgg-SIP	PspeB (SIP)
	0.96	3884.62	1610.58
	1.01	3700.00	1588.46
	0.96	6532.69	1644.23
	1.07	6577.88	1928.85
Mean	1.00	5173.80	1693.03
StddEv	0.05	1597.09	158.87

Fig 1C	SIP*			SIP*ΔpepO			SIP*Δrgg		
SIP	0	0.1	0.5	0	0.1	0.5	0	0.1	0.5
	1.00	18.25	116.16	0.95	45.25	304.44	458.25	481.04	580.04
	1.00	19.03	122.79	1.09	39.95	302.33	439.59	504.95	552.56
	1.000	20.706	119.218	0.93	28.50	155.99	468.24	519.09	521.51
	1.092	20.490	120.125	0.97	28.18	187.28	473.74	515.17	496.17
SIP	0	0.1	0.5	0	0.1	0.5	0	0.1	0.5
	0.98	17.89	113.88	0.93	44.37	298.47	449.27	471.60	568.66
	0.98	18.65	120.38	1.07	39.16	296.41	430.97	495.05	541.73
	0.98	20.30	116.88	0.91	27.94	152.93	459.06	508.91	511.29
	1.07	20.09	117.77	0.95	27.63	183.60	464.45	505.07	486.44
								1.10	1.17
Mean	1.00	19.23	117.23	0.96	34.77	232.85	450.94	495.16	527.03
StdEv	0.05	1.15	2.68	0.07	8.35	75.62	14.72	16.76	35.80

Fig 1G	A20	ΔropB	ΔropB+PropB
	1	0.617	2.281
	0.895	0.631	2.136
	1.000	0.129	3.188
	0.983	0.134	3.331
Fig 1G	A20	ΔropB	ΔropB+PropB
	1.03	0.64	2.35
	0.92	0.65	2.20
	1.03	0.13	3.29
	1.01	0.14	3.44
Mean	1.00	0.39	2.82
StdEv	0.05	0.29	0.63

Fig. 1D	SIP*			SIP*ΔpepO			SIP*Δrgg		
SCRA	0	0.1	0.5	0	0.1	0.5	0	0.1	0.5
	1.14	2.09	0.70	1.89	1.17	1.29	590.18	659.40	650.32
	0.88	1.68	1.33	1.59	1.85	1.23	650.32	677.93	598.41
	1.00	0.75	0.97	12.64	0.80	1.00	565.96	583.55	607.98
	0.86	0.96	0.99	0.00	1.17	1.16	543.16	572.72	559.63
SCRA	0	0.1	0.5	0	0.1	0.5	0	0.1	0.5
	1.17	2.16	0.72	1.94	1.20	1.33	608.43	679.79	670.43
	0.91	1.73	1.37	1.63	1.90	1.26	670.43	698.90	616.92
	1.03	0.77	0.99		0.82	1.03	583.47	601.60	626.78
	0.89	0.99	1.02		1.20	1.19	559.96	590.43	576.94
Mean	1.00	1.41	1.03	1.79	1.28	1.20	605.57	642.68	622.77
StdEv	0.13	0.64	0.27	0.22	0.45	0.13	47.55	54.64	38.39

Figure 1I	SIP*Δrgg-6h	SIP*Δrgg-7h	SIP*-6h	SIP*-7h	A20Δrgg-6h	A20Δrgg-7h	A20-6h	A20-7h
	0.10	0.14	0.00	0.00	0.12		1.07	2.68
	0.09	0.19	0.00	0.00	0.10	0.22	0.94	2.55
	0.08	0.17	0.00	0.00	0.08	0.16	1.07	1.61
	0.06	0.18	0.00	0.00	0.06	0.14	0.93	1.90
Mean	0.08	0.17	0.00	0.00	0.09	0.18	1.00	2.18
StdEV	0.02	0.02	0.00	0.00	0.03	0.04	0.08	0.51
speB	6 h	7 h	6 h	7 h	6 h	7 h	6 h	7 h
T-test	0.00	0.00			0.00	0.00		
	0.68	0.79					As 1	As 1
		0.00		0.19		0.02		0.00

Fig 2A	A20	ΔpepO	ΔcovR	ΔcovRΔpepO	
	1	4.599	0.940	12.769	
	0.895	4.857	0.914	13.052	
	1.000	4.991	1.336	20.714	
	0.983	5.273	1.274	19.830	
**Fig 2A**	**A20**	**ΔpepO**	**ΔcovR**	**ΔcovRΔpepO**	
	1.03	4.75	1.97	13.18	
	0.92	5.01	1.94	13.47	
	1.03	5.15	1.38	21.38	
	1.01	5.44	1.31	20.46	
**Mean**	1.00	5.09	1.65	17.12	
**StdEv**	0.05	0.29	0.35	4.40	

Fig 2B	ΔcovR-5h	ΔcovR-6h	ΔcovR-7h	ΔcovRΔrgg-5h	ΔcovRΔrgg-6h	ΔcovRΔrgg-7h	ΔcovRSIP*-5h	ΔcovRSIP*-6h	ΔcovRSIP*-7h
	1.04	278.20	594.28	6.36	7.86	13.69	0.01	0.02	0.01
	0.97	270.60	606.77	5.35	7.04	11.51	0.01	0.02	0.02
	1.04	179.15	353.36	4.27	6.06	7.59	0.01	0.02	0.01
	0.97	182.91	348.50	4.04	6.11	6.75	0.01	0.02	0.01
**Mean**	1.00	227.71	475.73	5.01	6.77	9.89	0.01	0.02	0.02
**StdEV**	0.04	54.02	144.21	1.07	0.86	3.28	0.00	0.00	0.00
				ΔcovRΔrgg	ΔcovRΔrgg	ΔcovRΔrgg	ΔcovR-SIP*	ΔcovR-SIP*	ΔcovR-SIP*

Fig 2D	5 h	6 h	7 h	5 h	6 h	7 h	5 h	6 h	7 h
	205.07	447.27	479.37	1.09	1.22	0.76	237.21	300.25	293.05
	215.27	378.72	463.04	0.91	1.14	0.70	240.52	278.20	269.66
	306.55	376.11	474.41	0.93	1.51	0.94	276.28	301.29	316.27
	330.84	331.99	477.71	1.08	1.72	1.04	292.04	303.38	293.05
**Mean**	264.44	383.52	473.63	1.00	1.40	0.86	261.51	295.78	293.01
**StdEv**	63.57	47.60	7.36	0.10	0.27	0.16	26.97	11.79	19.03
	**ΔcovRΔrgg**	**ΔcovRΔrgg**	**ΔcovRΔrgg**	**ΔcovR-SIP***	**ΔcovR-SIP***	**ΔcovR-SIP***	**ΔcovR-SIP*Δrgg**	**ΔcovR-SIP*Δrgg**	**ΔcovR-SIP*Δrgg**

Fig 3C	AP3				AP3ΔpepO			
SIP	0	0.1	0.5	1.0	0	0.1	0.5	1.0
20210817-1	1.00	1.84	5.82	2.27	19.03	10.85	89.88	65.80
	1.09	1.52	6.02	2.24	20.39	11.16	92.41	70.03
20210817-2	1.00	1.93	3.84	6.06	1.93	26.35	7.06	78.25
	1.04	1.93	3.66	7.11	1.92	26.17	7.73	78.25
20210822-1	1.00	1.17	5.82	9.25	1.78	12.82	54.95	92.41
	1.13	1.37	5.70	8.11	1.53	12.55	54.19	83.87
20210822-2	1.00	1.57	3.12	6.36	2.73	17.39	68.59	126.20
	0.91	1.75	2.95	7.11	3.01	17.15	78.79	113.80
SIP	0	0.1	0.5	1.0	0	0.1	0.5	1.0
	0.99	1.91	3.80	6.00	1.91	26.09	88.99	77.48
	1.03	1.91	3.62	7.04	1.90	25.91	91.50	77.48
	0.99	1.16	5.76	9.16	1.76	12.69	54.41	91.50
	1.12	1.35	5.64	8.03	1.51	12.43	53.65	83.04
	0.99	1.55	3.09	6.30	2.70	17.22	67.91	124.95
	0.90	1.74	2.92	7.04	2.98	16.98	78.01	112.67
Mean	1.00	1.60	4.14	7.26	2.13	18.55	72.41	94.52
StdEV	0.07	0.31	1.25	1.17	0.58	6.12	16.53	19.89

Fig 3C		SCRA (μM)			
speB	AP3+0.5 SIP	AP3+0	AP3+1	AP3ΔpepO+0	AP3+1
	1.00	0.43	0.49	0.72	0.40
	0.97	0.33	0.40	0.80	0.42
		0.28	0.32	0.37	1.22
		0.25	0.27	0.44	0.62
speB	0.5	0	1	0	1
	1.02	0.44	0.50	0.73	0.41
	0.99	0.34	0.41		0.43
		0.28	0.33	0.37	
		0.26	0.28	0.45	0.63
Mean	1.00	0.36	0.41	0.55	0.42
StdEv	0.02	0.08	0.09	0.25	0.01

Fig 3D	H280A	A20	T284A
ropB	0.13	0.98	0.97
	0.12	1.01	1.02
	0.07	0.85	1.26
	0.24	1.02	1.28
	0.24	0.97	1.30
	0.09	1.15	1.39
	0.13	1.07	2.07
	0.13	0.95	2.08
Mean	0.14	1.00	1.42
StdEv	0.06	0.09	0.43

Fig 3G	A20	D53A	D53AΔropB
	1	0.001	0.693
	0.895	0.001	0.764
	1.000	0.002	1.034
	0.983	0.003	0.98
Fig 3G	A20	D53A	D53AΔropB
		1.00	
		1.00	617.07
		1.00	541.77
		1.00	349.70
Mean		1.00	502.85
StdEv		0.00	137.87

Fig 3F	H280A	H280AΔrgg
speB	1.26	429.89
	1.31	421.61
	0.74	355.77
	0.69	404.17
Mean	1.00	402.86
StdEv	0.33	33.17

Fig 4D-speB	A20	ΔropB	SIP*	SIP*ΔropB
	1	0.617	0.001	0.408
	0.895	0.631	0.001	0.396
	1.000	0.129	0.000	0.717
	0.983	0.134	0.000	0.761
**Fig 4D-speB**	**A20**	**ΔropB**	**SIP***	**SIP*ΔropB**
	1.032	0.637	0.001	0.421
	0.924	0.651	0.002	0.409
	1.032	0.133	0.000	0.740
	1.014	0.138	0.000	0.785
**Mean**	1.000	0.390	0.001	0.589
**StdEv**	0.052	0.294	0.001	0.202

Fig 4D	A20	ΔropB	SIP*	SIP*ΔropB
**spy1733-1734**	1.000	0.115	0.001	0.176
	1.003	0.103	0.001	0.152
	1.000	0.564	0.007	0.276
	0.741	0.511	0.007	0.308
**Fig 5D-1733**	**A20**	**ΔropB**	**SIP***	**SIP*ΔropB**
	1.068	0.122	0.001	0.188
	1.072	0.111	0.001	0.162
	1.068	0.603	0.007	0.294
	0.792	0.546	0.008	0.329
**Mean**	1.000	0.346	0.004	0.244
**StdEv**	0.139	0.266	0.004	0.081

Fig 4D	A20	ΔropB	SIP*	SIP*ΔropB
**prsA**	1.000	0.177	0.052	0.338
	0.996	0.178	0.044	0.308
	1.000	0.869	0.257	0.531
	0.974	0.899	0.236	0.562
**Fig 5D-prsA**	**A20**	**ΔropB**	**SIP***	**SIP*ΔropB**
	1.008	0.178	0.053	0.341
	1.004	0.179	0.044	0.310
	1.008	0.876	0.259	0.535
	0.982	0.906	0.238	0.567
**Mean**	1.000	0.535	0.149	0.438
**StdEv**	0.013	0.412	0.116	0.131

Fig 5AB (upper panel)	A20	SIP	SIPΔropB
M5005_Spy1176	0.228404	1.02329	34.927769
M5005_Spy1416	65.983047	30.363028	0.0001
M5005_Spy1426	54.295586	18.731243	0.0001

Fig 5C	A20		ΔropB		SIP*		SIP*ΔropB	
ropB	pH 7.5	pH 6.0	pH 7.5	pH 6.0	pH 7.5	pH 6.0	pH 7.5	pH 6.0
Exp-1	1.013	0.957	0.000	0.000	1.054	0.774	0.000	0.000
	0.988	1.050	0.000	0.000	0.942	1.089	0.000	0.000
Exp-2	1.000	0.723	0	0	1.000	1.172	0	0
	1.091	0.767	0	0	1.091	1.173	0	0
Mean	1.023	0.874	0.000	0.000	1.022	1.052	0.000	0.000
StdEv	0.047	0.155	0.000	0.000	0.065	0.190	0.000	0.000

Fig 5C	A20		ΔropB		SIP*		SIP*ΔropB	
speB	pH 7.5	pH 6.0	pH 7.5	pH 6.0	pH 7.5	pH 6.0	pH 7.5	pH 6.0
Exp-1	0.954	4,761.905	384.986	288.860	0.351	0.465	366.139	364.303
	1.046	6,049.590	446.558	306.712	0.395	0.501	442.124	301.501
Exp-2	1.000	2,971.206	264.107	211.264	0.222	0.365	276.939	211.264
	0.833	3,212.016	249.171	213.318	0.379	0.502	280.088	213.318
Mean	0.958	4,248.679	336.205	255.039	0.337	0.458	341.323	272.597
StdEv	0.092	1,439.131	95.447	49.903	0.079	0.064	78.892	74.210

Fig 5D	A20		ΔropB		SIP*		SIP*ΔropB	
spy1176	pH 7.5	pH 6.0	pH 7.5	pH 6.0	pH 7.5	pH 6.0	pH 7.5	pH 6.0
Exp-1	1.148	1.026	1.432	0.820	1.160	0.696	36.057	62.926
	0.852	1.266	1.818	0.858	1.014	0.804	32.973	54.093
Exp-2	1.000	1.579	0.589	1.527	1.186	2.812	141.640	151.762
	0.433	0.941	1.067	1.362	1.316	1.321	162.312	147.902
Mean	0.858	1.203	1.227	1.142	1.169	1.408	93.246	104.171
StdEv	0.308	0.286	0.524	0.356	0.124	0.975	68.351	52.872

Fig 5E	A20		ΔropB		SIP*		SIP*ΔropB	
spy1416	pH 7.5	pH 6.0	pH 7.5	pH 6.0	pH 7.5	pH 6.0	pH 7.5	pH 6.0
Exp-1	1.000	5.465	0.793	4.474	1.027	4.717	0.000	0.000
	1.034	5.720	0.936	4.366	1.038	4.802	0.000	0.006
Exp-2	1.000	4.127	0.783	3.491	0.748	7.100	0	0.073
	1.291	3.839	0.722	5.130	0.957	6.962	0.002	0
Mean	1.081	4.788	0.808	4.365	0.943	5.895	0.001	0.020
StdEv	0.141	0.942	0.091	0.674	0.134	1.313	0.001	0.036

Fig 5E	A20		ΔropB		SIP*		SIP*ΔropB	
spy1426	pH 7.5	pH 6.0	pH 7.5	pH 6.0	pH 7.5	pH 6.0	pH 7.5	pH 6.0
Exp-1	1.000	6.842	0.713	5.753	0.898	6.150	0.000	0.000
	0.817	6.544	0.935	5.205	0.877	5.635	0.000	0.000
Exp-2	1.000	4.453	0.599	5.298	0.611	6.777	0	0
	1.285	4.288	0.719	5.577	0.947	7.850	0	0
Mean	1.026	5.532	0.742	5.458	0.833	6.603	0.000	0.000
StdEv	0.194	1.348	0.140	0.252	0.151	0.953	0.000	0.000

Fig 5A lower panel	A20	SIP*	SIP*ΔropB
spy1176	0.899	0.702	45.743
	0.899	0.652	43.797
	1.228	0.724	44.748
	0.899	0.665	124.973
	1.102	0.503	115.234
	0.899	1.351	103.720
	1.162	0.906	103.144
Mean	1.01	0.79	83.05
StdEv	0.147	0.276	36.570

Fig 5B lower panel	A20	SIP*	SIP*ΔropB
spy1416	1.01	0.67	-0.58
	1.15	0.83	-0.27
	1.01	0.57	-0.82
	0.93	0.52	-0.94
	1.01	0.89	-0.16
	1.00	0.69	-0.53
	1.01	0.85	-0.24
	0.87	0.89	-0.18
Mean	1.00	0.74	-0.46
StdEv	0.08	0.15	0.30

Fig 5B lower panel	A20	SIP*	SIP*ΔropB
spy1426	1.00	0.87	-0.21
	0.99	0.86	-0.21
	1.00	0.61	-0.72
	1.03	0.58	-0.80
	1.00	0.73	-0.46
	0.97	0.76	-0.39
Mean	1.00	0.73	-0.46
StdEv	0.02	0.12	0.25

Fig S2-AB (upper panel)	A20	SIP	SIPΔropB
M5005_Spy1189	0.320708	1.14662	39.911957
adh2	115.491882	29.519796	23.897396
M5005_Spy0023	82.782333	23.002945	10.393085

Fig S2 C	A20		ΔropB		SIP*		SIP*ΔropB	
spy1189	pH 7.5	pH 6.0	pH 7.5	pH 6.0	pH 7.5	pH 6.0	pH 7.5	pH 6.0
Exp-1	1.000	0.572	1.183	0.895	0.537	0.655	19.935	35.153
	0.671	1.248	1.226	0.617	1.092	0.775	20.048	30.576
Exp-2	1.000	2.955	1.320	2.512	1.546	3.008	136.791	174.885
	2.624	3.291	0.874	3.375	1.619	2.874	148.748	119.651
Mean	1.324	2.016	1.151	1.850	1.199	1.828	81.380	90.066
StdEv	0.881	1.314	0.193	1.316	0.499	1.287	71.054	69.819

Fig S2 C	A20		ΔropB		SIP*		SIP*ΔropB	
adh2	pH 7.5	pH 6.0	pH 7.5	pH 6.0	pH 7.5	pH 6.0	pH 7.5	pH 6.0
Exp-1	1.000	0.386	0.986	0.535	0.824	0.473	1.096	0.735
	1.029	0.442	0.981	0.547	0.872	0.462	0.902	0.915
Exp-2	1.000	0.444	0.998	0.497	0.922	0.546	1.432	0.952
	1.092	0.431	1.038	0.569	0.924	0.497	1.553	0.858
Mean	1.030	0.426	1.001	0.537	0.885	0.495	1.246	0.865
StdEv	0.043	0.027	0.026	0.030	0.048	0.037	0.300	0.095

Fig S2 C	A20		ΔropB		SIP*		SIP*ΔropB	
spy0023	pH 7.5	pH 6.0	pH 7.5	pH 6.0	pH 7.5	pH 6.0	pH 7.5	pH 6.0
Exp-1	1.000	6.682	1.309	5.966	1.146	6.536	1.367	3.884
	1.297	5.700	1.010	5.956	1.203	6.325	0.998	4.003
Exp-2	1.000	4.313	0.703	5.111	0.752	3.569	1.929	5.209
	0.978	4.154	0.793	5.537	0.947	3.310	2.012	5.040
Mean	1.069	5.212	0.954	5.643	1.012	4.935	1.577	4.534
StdEv	0.152	1.201	0.269	0.407	0.206	1.732	0.481	0.687

Fig S2 A lower panel	A20	SIP*	SIP*ΔropB
spy1189	1.007	0.428	15.142
	1.223	0.513	15.175
	1.007	0.491	16.824
	0.927	0.568	15.191
	1.007	0.794	32.066
	0.956	0.537	33.325
Mean	1.00	0.56	21.29
StdEv	0.104	0.126	8.869

Fig S2B lower panel	A20	SIP*	SIP*ΔropB
adh2	1.02	0.56	0.71
	0.99	0.54	0.62
	0.99	0.66	0.94
	0.99	0.78	0.97
Mean	1.00	0.64	0.81
StdEv	0.02	0.11	0.18

Fig S2B lower panel	A20	SIP*	SIP*ΔropB
spy0023	1.00	0.77	0.78
	1.05	0.81	0.86
	1.00	1.00	0.76
	0.95	1.10	0.75
Mean	1.00	0.92	0.79
StdEv	0.04	0.16	0.05

Fig S3A	A20	ΔcovR
**speB**	1.06	1.59
	1.13	2.52
	1.18	2.59
**Mean**	1.12	2.24
**StdEv**	0.06	0.56

Fig S3B	A20	ΔcovR
**ropB**	0.99	1.28
	1.01	
	0.90	2.41
	1.10	2.61
**Mean**	1.00	2.10
**StdEv**	0.08	0.72
