# Supplementary material for: RopB represses the transcription of speB in the absence of SIP in group A Streptococcus
Source: Life Sci Alliance. 2023 Mar 31;6(6):e202201809. doi: 10.26508/lsa.202201809 (PMC10071013; doi:10.26508/lsa.202201809)
Supplement: Supplementary file 3 [file LSA-2022-01809_TableS1.docx]

**Supplementary Table S1.** Significantly upregulated and downregulated genes (*q* value < 0.05) in the *SIP** mutant compared to those in the wild-type A20 strain.

| **Spy number** | **Locus tag** | **Fold change** | ***q* value** | **Annotation** |
| --- | --- | --- | --- | --- |
| **M5005_Spy1733** | *M5005_Spy1733* | 0.00 | 1.76E-08 | hypothetical protein |
| **M5005_Spy1734** | *spi* | 0.02 | 2.08E-20 | streptopain protease inhibitor |
| **M5005_Spy1735** | *speB* | 0.02 | 7.23E-42 | streptococcal pyrogenic exotoxin B |
