# Supplementary material for: RopB represses the transcription of speB in the absence of SIP in group A Streptococcus
Source: Life Sci Alliance. 2023 Mar 31;6(6):e202201809. doi: 10.26508/lsa.202201809 (PMC10071013; doi:10.26508/lsa.202201809)
Supplement: Supplementary file 4 [file LSA-2022-01809_TableS2.docx]

**Supplementary Table S2.** The significantly upregulated and downregulated genes (*q* value < 0.05) in the *SIP**/∆*ropB* mutant compared to those in the *SIP** mutant.

| **Spy number** | **Locus tag** | **Fold change** | ***q* value** | **Annotation** |
| --- | --- | --- | --- | --- |
| **M5005_Spy1737** | *rgg* | 0.00 | 2.63E-12 | transcriptional regulator |
| **M5005_Spy1735** | *speB* | 8.79 | 2.42E-04 | streptococcal pyrogenic exotoxin B |
| **M5005_Spy1734** | *spi* | 12.11 | 3.73E-02 | streptopain protease inhibitor |
| **M5005_Spy1733** | *M5005_Spy1733* | 1082983.20 | 4.71E-03 | hypothetical protein |
| **M5005_Spy1566** | *recD* | 10.06 | 3.35E-03 | exodeoxyribonuclease V alpha chain |
| **M5005_Spy1540** | *endoS* | 5.06 | 3.71E-02 | endo-beta-N-acetylglucosaminidase F2 precursor |
| **M5005_Spy1530** | *M5005_Spy1530* | 15.36 | 3.68E-06 | putative Fe3+-siderophore transport protein |
| **M5005_Spy1525** | *M5005_Spy1525* | 11.11 | 1.66E-02 | ABC transporter ATP-binding protein |
| **M5005_Spy1435** | *M5005_Spy1435* | 0.00 | 3.23E-03 | phage scaffold protein |
| **M5005_Spy1426** | *M5005_Spy1426* | 0.00 | 7.40E-10 | phage protein |
| **M5005_Spy1425** | *M5005_Spy1425* | 0.00 | 4.97E-02 | phage protein |
| **M5005_Spy1424** | *M5005_Spy1424* | 0.00 | 1.40E-04 | phage endopeptidase |
| **M5005_Spy1421** | *M5005_Spy1421* | 0.00 | 1.33E-03 | phage infection protein |
| **M5005_Spy1416** | *M5005_Spy1416* | 0.00 | 9.16E-04 | phage-associated cell wall hydrolase |
| **M5005_Spy1407** | *M5005_Spy1407* | 41.30 | 1.12E-02 | esterase |
| **M5005_Spy1399** | *M5005_Spy1399* | 14.69 | 1.95E-02 | PTS system, galactose-specific IIC component |
| **M5005_Spy1395** | *lacD.1* | 24.29 | 3.99E-02 | tagatose-bisphosphate aldolase |
| **M5005_Spy1378** | *M5005_Spy1378* | 17.57 | 1.47E-02 | NADH peroxidase |
| **M5005_Spy1377** | *M5005_Spy1377* | 18.22 | 8.52E-03 | trans-acting positive regulator |
| **M5005_Spy1348** | *M5005_Spy1348* | 20.69 | 1.12E-02 | D-beta-hydroxybutyrate permease |
| **M5005_Spy1339** | *priA* | 14.33 | 8.06E-04 | primosomal protein N' |
| **M5005_Spy1327** | *comFA* | 27.21 | 1.14E-02 | COMF operon protein 1 |
| **M5005_Spy1176** | *M5005_Spy1176* | 34.13 | 4.47E-02 | phage infection protein |
