# Supplementary material for: RopB represses the transcription of speB in the absence of SIP in group A Streptococcus
Source: Life Sci Alliance. 2023 Mar 31;6(6):e202201809. doi: 10.26508/lsa.202201809 (PMC10071013; doi:10.26508/lsa.202201809)
Supplement: Supplementary file 5 [file LSA-2022-01809_TableS3.docx]

**Supplementary Table S3.** The significantly upregulated and downregulated genes (*q* value < 0.05) in the *SIP**/∆*ropB* mutant compared to those in the wild-type strain.

| **Spy number** | **Locus tag** | **Fold change** | ***q* value** | **Annotation** |
| --- | --- | --- | --- | --- |
| **M5005_Spy1773** | *M5005_Spy1773* | 776207 | 9.17E-04 | formiminotetrahydrofolate cyclodeaminase |
| **M5005_Spy1737** | *rgg* | 0.00 | 1.70E-08 | transcriptional regulator |
| **M5005_Spy1735** | *speB* | 0.15 | 3.40E-16 | streptococcal pyrogenic exotoxin B |
| **M5005_Spy1734** | *spi* | 0.22 | 1.38E-03 | streptopain protease inhibitor |
| **M5005_Spy1467** | *int.3* | 0.00 | 1.08E-02 | integrase |
| **M5005_Spy1453** | *M5005_Spy1453* | 0.00 | 1.72E-03 | phage protein |
| **M5005_Spy1450** | *M5005_Spy1450* | 0.00 | 5.88E-06 | phage-encoded DNA polymerase |
| **M5005_Spy1449** | *M5005_Spy1449* | 0.00 | 1.27E-07 | DNA primase |
| **M5005_Spy1447** | *M5005_Spy1447* | 0.00 | 9.17E-04 | phage-related DNA helicase |
| **M5005_Spy1444** | *M5005_Spy1444* | 0.00 | 3.62E-02 | adenine-specific methyltransferase |
| **M5005_Spy1440** | *M5005_Spy1440* | 0.00 | 1.07E-03 | terminase large subunit |
| **M5005_Spy1439** | *M5005_Spy1439* | 0.00 | 2.74E-04 | portal protein |
| **M5005_Spy1438** | *M5005_Spy1438* | 0.00 | 2.64E-04 | phage protein |
| **M5005_Spy1435** | *M5005_Spy1435* | 0.00 | 2.85E-04 | phage scaffold protein |
| **M5005_Spy1434** | *M5005_Spy1434* | 0.00 | 1.72E-03 | phage protein |
| **M5005_Spy1429** | *M5005_Spy1429* | 0.00 | 4.19E-02 | phage protein |
| **M5005_Spy1426** | *M5005_Spy1426* | 0.00 | 6.31E-21 | phage protein |
| **M5005_Spy1425** | *M5005_Spy1425* | 0.00 | 6.31E-04 | phage protein |
| **M5005_Spy1424** | *M5005_Spy1424* | 0.00 | 1.01E-10 | phage endopeptidase |
| **M5005_Spy1423** | *M5005_Spy1423* | 0.00 | 2.85E-04 | hyaluronoglucosaminidase |
| **M5005_Spy1422** | *M5005_Spy1422* | 0.00 | 4.12E-02 | phage protein |
| **M5005_Spy1421** | *M5005_Spy1421* | 0.00 | 8.30E-09 | phage infection protein |
| **M5005_Spy1419** | *M5005_Spy1419* | 0.00 | 2.73E-02 | phage protein |
| **M5005_Spy1416** | *M5005_Spy1416* | 0.00 | 2.73E-06 | phage-associated cell wall hydrolase |
| **M5005_Spy1415** | *sdaD2* | 0.00 | 1.91E-03 | phage-encoded streptodornase |
| **M5005_Spy1189** | *M5005_Spy1189* | 124.45 | 2.80E-02 | phage terminase |
| **M5005_Spy1176** | *M5005_Spy1176* | 152.92 | 6.71E-03 | phage infection protein |
| **M5005_Spy0518** | *M5005_Spy0518* | 0.00 | 4.60E-02 | oligohyaluronate lyase |
| **M5005_Spy0039** | *adh2* | 0.21 | 2.80E-02 | alcohol dehydrogenase/acetaldehyde dehydrogenase (acetylating) |
| **M5005_Spy0028** | *M5005_Spy0028* | 0.00 | 2.74E-04 | autolysin |
| **M5005_Spy0023** | *M5005_Spy0023* | 0.13 | 9.56E-04 | phosphoribosylformylglycinamidine synthase |
