# Supplementary material for: RopB represses the transcription of speB in the absence of SIP in group A Streptococcus
Source: Life Sci Alliance. 2023 Mar 31;6(6):e202201809. doi: 10.26508/lsa.202201809 (PMC10071013; doi:10.26508/lsa.202201809)
Supplement: Supplementary file 6 [file LSA-2022-01809_TableS4.docx]

**Supplementary Table S4**. Primers used in this study.

| **Primer** | **Use** | **Sequence (5’-3’)*^a^*** | **Reference or source** |
| --- | --- | --- | --- |
| ropB-F-05 | Construction | ggcatgcaggctttttcctggctgatt | This study |
| ropB-F-04 | Construction | ggcatgcaggaggtcgtgaccaaaaaag | This study |
| ropB-EcoRV-F | Construction | attcggatatctgaggctctttaaaaagcta | This study |
| ropB-EcoRV-R | Construction | attcggatatcatgtcaagccttcctagttg | This study |
| PepO-SacII-F | Construction | tccccgcggtaattaggtagttgataaacc | (Shi et al, 2022) |
| PepO-SacII-R | Construction | tccccgcggcattcgtatctccttatatca | (Shi et al, 2022) |
| PepO-BamHI-F | Construction | gcgggatcccgagacatcaatgtcgaaaca | (Shi et al, 2022) |
| PepO-BamHI-R | Construction | gcgggatccgaatctcgtgcaatggttgat | (Shi et al, 2022) |
| RopB-F-3 | qPCR | Tttgaatgccgaaacatagaaggtt | (Chiang-Ni et al, 2016) |
| RopB-R-2 | qPCR | ctaataacaccttgaccaaggcaaa | (Chiang-Ni et al, 2016) |
| Orf-1-F | Construction | gttaaaaggaggcgcctactTAgtggttattgttactatttttg | (Do et al, 2017) |
| Orf-1-R | Construction | caaaaatagtaacaataaccacTAagtaggcgcctccttttaac | (Do et al, 2017) |
| SIP-F-1 | Construction | gcgggatccggtcaatagccagatgcgata | (Do et al, 2017) |
| SIP-R-1 | Construction | gcgggatcctcgtgatagggtccacaaca | (Do et al, 2017) |
| speB-2-F | qPCR | tgcctacaacagcactttgg | (Chiang-Ni et al, 2016) |
| speB-2-R | qPCR | ggtaaagtaggcggacatgc | (Chiang-Ni et al, 2016) |
| M5005_Spy1176-F | qPCR | tgtttgatagtcgtattagtgg | This study |
| M5005-Spy1176-R | qPCR | ttgagatagcgttgagtagtt | This study |
| M5005_Spy1416-F | qPCR | tacctttacgcttgatgttgt | This study |
| M5005_Spy1416-R | qPCR | cagtgtctccttgtttgtagc | This study |
| M5005_Spy1426-F | qPCR | cgctatttgggtattctgct | This study |
| M5005_Spy1426-R | qPCR | atctaaacttcctgtggttatgg | This study |
| M5005_Spy1189-F | qPCR | gaggcacaagaatacacatc | This study |
| M5005_Spy1189-R | qPCR | gcgttccacacataatagtc | This study |
| adh2-F | qPCR | agtagaaccagatccagaca | This study |
| adh2-R | qPCR | tcgtagaagagccacataac | This study |
| M5005_Spy0023-F | qPCR | cagttgagattgaagtagatgttg | This study |
| M5005_Spy0023-R | qPCR | atttctgttgggtgattgtg | This study |
| gyrA-F-3 | qPCR | cgtcgtttgactggtttgg | (Chiang-Ni et al, 2016) |
| gryA-R-3 | qPCR | ggcgtgggttagcgtattta | (Chiang-Ni et al, 2016) |
| P1-del-BamHI-F | Construction | ccttctggatccatgtttcggcattca | This study |
| P1-del-BamHI-R | Construction | aggcttggatcctttcctggctgatt | This study |
| P1-SacII-F-1 | Construction | tacgtaccgcggacaaaataatgggtta | This study |
| P1-SacII-R-1 | Construction | gacatcccgcggaactaggaaggcttga | This study |
| P1-SacII-F-2 | Construction | ataggtccgcgggcataaggtcaatagc | This study |
| P1-SacII-R-2 | Construction | gtatttccgcggttgacatcaactagga | This study |
| P1-SacII-F-3 | Construction | ctaataccgcggcgtaacaaaataatgg | This study |
| P1-SacII-R-3 | Construction | tagacaccgcgggttgataaaactatcg | This study |
| PspeB-SacI-F-2 | Construction | cgagctcgtttcaccaatttccatatg | This study |
| PspeB-SacI-R-2 | Construction | cgagctctttttttatacctct ttcaa | This study |
| PspeB-Pdel-BamHI-R | Construction | ccgggatccttagcgtcaaatgatcc | This study |
| PspeB-Pdel-SacI | Construction | tccgagctcatgaataaaaagaaattagg | This study |
| PspeB-P2del-SacI | Construction | tccgagctctcatgtgagcctaattggtt | This study |

*^a^*Underlining indicates restriction enzyme sites; uppercase letters denote mutated nucleotides.
